# High Tibial Osteotomy Is Associated with Improvements in Both Knee and Ankle Alignment in Medial Compartment Osteoarthritis

**DOI:** 10.3390/jcm15010315

**Published:** 2026-01-01

**Authors:** Umut Oktem, Muhammed Cihan Dastan, Hanife Avci, Mustafa Bulut, Gulfem Ezgi Ozaltin, Durmus Ali Ocguder, Osman Tecimel, Izzet Bingol

**Affiliations:** 1 Department of Orthopaedics and Traumatology, Ankara City Hospital, 06800 Ankara, Türkiye; drumutoktem@gmail.com (U.O.); drcihan@yahoo.com (M.C.D.); 2 Department of Biostatistics, Faculty of Medicine, Hacettepe University, 06100 Ankara, Türkiye; hanife.avci@hacettepe.edu.tr; 3 Department of Orthopaedics and Traumatology, Aksaray University Training and Research Hospital, 50300 Aksaray, Türkiye; drbulut01@gmail.com; 4 Department of Physiotherapy and Rehabilitation, Inonu University, 44280 Malatya, Türkiye; 5 Department of Orthopaedics and Traumatology, Ankara Yıldırım Beyazıt University, 06010 Ankara, Türkiye; aliocguder@yahoo.com (D.A.O.); dr.izzetbingol@hotmail.com (I.B.); 6 Department of Orthopaedics and Traumatology, Memorial Health Group Ankara Hospital, 06520 Ankara, Türkiye; drtecimel@gmail.com

**Keywords:** tibial osteotomy, ankle alignment, knee osteoarthritis, lower-extremity biomechanics, talar tilt

## Abstract

**Introduction**: Medial compartment knee osteoarthritis (OA) is characterized by varus deformity. A medial open-wedge high tibial osteotomy (MOWHTO), frequently invoked in the treatment of this deformity, affects the knee as well as the ankle joints. This study aims to evaluate the radiological and clinical effects of a MOWHTO on the ankle joint. **Materials and Methods**: A retrospective analysis was conducted with data from 110 patients (mean age: 52 years; 74.5% female) who underwent a MOWHTO between 2020 and 2023. Radiographic assessments were conducted both preoperatively and one year after surgery using full-length weight-bearing radiographs. The measurements included several alignment parameters such as the hip–knee–ankle angle (HKA), medial proximal tibial angle (MPTA), joint line convergence angle (JLCA), lateral distal tibial angle (LDTA), and talar tilt. Clinical outcomes were assessed using the Lysholm knee score and the American Orthopedic Foot and Ankle Society (AOFAS) ankle score. **Results**: While changes in the LDTA demonstrated a small effect size (d = 0.225), moderate-to-large effect sizes were observed in key alignment parameters (MPTA (d = 0.838), the JLCA (d = 0.798), and talar tilt (d = 0.752)), all of which showed statistically significant differences indicative of a correction in the joint alignment of potential clinical significance. Median Lysholm and AOFAS scores at one year were 90 and 100, respectively, indicating favorable clinical outcomes. No significant difference in outcomes was observed based on the amount of correction. **Conclusions**: An MOWHTO not only restores knee alignment but also significantly improves ankle alignment in the coronal plane. These findings suggest that an MOWHTO is associated with the restoration of knee alignment and with improvements in ankle alignment in the coronal plane.

## 1. Introduction

Medial compartment knee osteoarthritis (OA) is a degenerative joint disease characterized by varus deformity, and its increasing frequency, particularly in young and active individuals, is raising alarm. By affecting the overall alignment of the knee, the deformity also influences the mechanical axis of the lower extremity, resulting in changes in load distribution and alignment in distal joints, such as the ankle and subtalar joints [1].

A medial open-wedge high tibial osteotomy (MOWHTO), used in the treatment of varus deformity in the young and active population, aims to reduce the load on the medial compartment and alleviate symptoms by shifting the mechanical axis of the knee joint laterally [2]. Following this corrective procedure, changes in the mechanical axis of the lower extremity may also result in realignments in the coronal plane at the ankle joint [3]. The recent literature suggests that lateralization of the coronal lower-limb axis may influence ankle-joint line orientation, load distribution, and alignment [4]. Compensatory changes occurring at the ankle, subtalar joint, and foot level after total knee arthroplasty (TKA) have been extensively investigated in many studies [5,6,7]. With the correction of varus deformity in the knee following TKA, angular adaptations and load distribution changes have been observed in the ankle and subtalar joints, and these changes have been reported to be the potential cause of pain, functional loss, or secondary osteoarthritis in the ankle [8]. In contrast, studies investigating changes in the ankle joint following a MOWHTO are limited in number, and there is no consensus in the literature on this topic [9,10]. Furthermore, recent findings related to the epiligament theory indicate that ligamentous structures surrounding the knee possess distinct biological characteristics associated with adaptation and healing [11]. This concept suggests that soft-tissue responses to altered loading conditions may contribute to alignment changes beyond purely mechanical effects, providing an additional rationale for investigating distal joint adaptations after a MOWHTO.

Some studies report significant changes in tibial plafond inclination, talar tilt, or ankle joint line angle after a MOWHTO; however, the impact of these changes on clinical outcomes remains controversial [12,13,14]. The aim of this study is to evaluate the clinical and radiological effects of a MOWHTO performed for medial compartment knee osteoarthritis on the ankle joint as well as the potential effect of on ankle alignment. The primary outcome was defined as a radiological change in ankle alignment (LDTA and talar slope). Secondary outcomes included radiological knee alignment parameters (HKA, MPTA, JLCA) and 1-year postoperative clinical knee and ankle scores (Lysholm and AOFAS), along with subgroup comparisons according to the magnitude of correction (<10 mm vs. ≥10 mm).

## 2. Materials and Methods

### 2.1. Patient Selection

Ethical approval was obtained from the relevant institutional ethics committee (28 August 2024). The study population consisted of patients aged 18 to 60 years who had undergone a MOWHTO as a treatment for osteoarthritis primarily affecting the medial compartment of the knee between 2020 and 2023. All participants had a postoperative follow-up period of at least one year.

Patients who met at least one of the following criteria were excluded from the study: history of lower-extremity fracture, presence of rheumatologic diseases (e.g., rheumatoid arthritis, ankylosing spondylitis), and history of previous surgery involving the hip, knee, or ankle.

### 2.2. Preoperative Assessment and Surgical Procedure

During surgical planning, the correction angle was calculated based on the principle of shifting the mechanical loading axis to the 62% lateral point of the tibial plateau (Fujisawa point). Postoperative stabilization was achieved using a 4.5 mm locking compression plate, and no correction exceeding 14 mm was performed in any patient. Additionally, bone grafting was not used in any case. All surgeries were performed using a standard technique by the same orthopedic and traumatology surgical team.

A standardized rehabilitation protocol was applied to all patients postoperatively. On the first postoperative day, patients started exercises to maintain an active knee range of motion. Patients were mobilized with partial weight bearing until the second week and then transitioned to full weight bearing thereafter.

### 2.3. Clinical and Radiological Assessment

Demographic data and radiological measurements of the patients were systematically recorded. Radiological evaluation included preoperative long-leg weight-bearing anteroposterior radiographs, from which the hip–knee–ankle angle (HKA), joint line convergence angle (JLCA), medial proximal tibial angle (MPTA), lateral distal tibial angle (LDTA), and talar tilt angle were measured. The same parameters were remeasured using the long-leg weight-bearing radiographs obtained at the 1-year postoperative follow-up appointment and recorded using the evaluation form.

For clinical evaluation, the Lysholm score was used to assess knee function, “American Orthopedic Foot and Ankle Society Score” (AOFAS) was used to assess ankle function at the 1-year follow-up appointment. All radiological measurements and clinical evaluations were conducted by an experienced orthopedic and traumatology specialist.

### 2.4. Statistical Analysis

Statistical analyses were performed utilizing the open-source R software (version 4.4.1) and the SPSS statistical package for Windows (Version 23.0, Chicago, IL, USA). Normality of the data was assessed using the Shapiro–Wilk test, and variance homogeneity was tested using Levene’s test. Descriptive statistics were presented as the mean and standard deviation, median (interquartile range, IQR), and frequencies (percentages). Whether there was an association between categorical variables was examined using Yates’s continuity correction chi-square test and Fisher–Freeman–Halton tests. The differences between pre–post measurements were examined using the paired-sample *t*-test or Wilcoxon test, as appropriate. The Mann–Whitney U test was used to examine whether there were differences in numerical measurements between the <10 mm correction and ≥10 mm correction groups, as the normal distribution assumption was not met. Effect size ($r=z/\sqrt{n}$) was calculated for the Mann–Whitney U test and the Wilcoxon test. Cohen’s d effect size was calculated for the paired-sample *t*-test. For chi-square tests, Phi and Cramer’s V effect sizes were reported. The relationship between numerical variables was examined with the Spearman rank correlation coefficient. Intra-observer reliability was examined using intraclass correlation coefficients (ICCs) and confidence intervals. For the relationship between numerical variables, the “metan” package was used [15]. A *p*-value of less than 5% was considered to be statistically significant.

## 3. Results

A total of 110 patients were included in the study with a mean age of 52 (11) years. Among the participants, 82 (74.5%) were female. According to the Kellgren–Lawrence grading system, preoperatively, 36 patients (32.7%) were classified as grade 2 (Table 1).

Radiological evaluations performed before and after surgery showed statistically significant changes in all parameters. The median medial proximal tibial angle (MPTA) increased from 83° preoperatively to 87.4° postoperatively (*p* < 0.001). The mean hip–knee–ankle angle (HKA) increased from 171.44° ± 3.35 to 177.11° ± 2.32, which was also statistically significant (*p* < 0.001). The median joint line convergence angle (JLCA) decreased significantly from 3.18° to 1.95° (*p* < 0.001). The median lateral distal tibial angle (LDTA) in the ankle plane increased from 87.25° to 88.14° (*p* = 0.018). Additionally, a significant improvement was observed in the talar tilt angle, with the median value decreasing from 0.85° to 0.70° postoperatively (*p* < 0.001) (Table 2).

**Figure 1:**
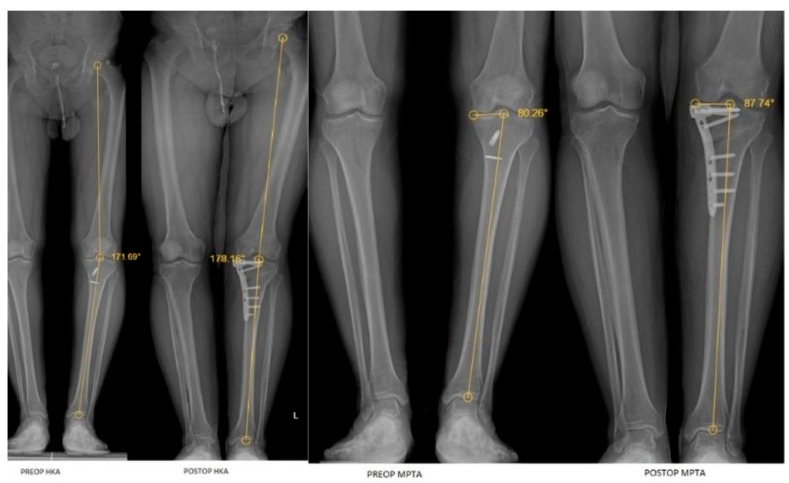
Preoperative and postoperative full-length lower-extremity radiographs showing knee and lower limb alignment measurements, including hip–knee–ankle angle (HKA) and medial proximal tibial angle (MPTA).

**Figure 2:**
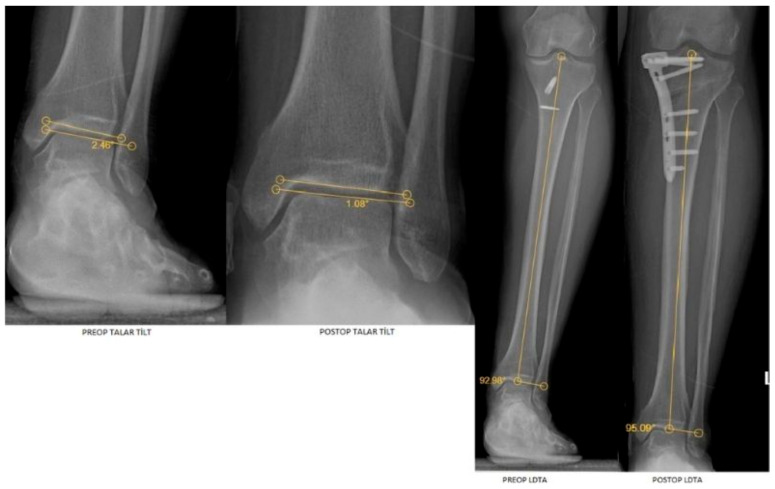
Preoperative and postoperative radiographic images demonstrating ankle joint measurements, including talar tilt and lateral distal tibial angle (LDTA).

Preoperative and postoperative radiographic measurements of the ankle and knee joints are presented in Figure 1 and Figure 2.

At the 1-year postoperative follow-up appointment, functional scores were assessed. The median Lysholm knee score was 90, and the median AOFAS ankle score was 100. No significant differences were found in Lysholm and AOFAS scores between the patient groups divided by correction amount (<10 mm and ≥10 mm), with *p*-values of 0.880 and 0.861, respectively.

Similarly, when comparing the two groups, the percentage change in JLCA did not reveal any statistically significant differences (–36.26 vs. –37.07, *p* = 0.785), percentage change in LDTA (0.55 vs. 0.65, *p* = 0.606), or percentage change in talar tilt (–13.03 vs. –9.11, *p* = 0.204) (Table 3).

A significant positive correlation was found between the amount of correction and the change in the HKA (r = 0.48, *p* < 0.001). A significant negative correlation was also observed between the amount of correction and the change in the JLCA (r = –0.32, *p* < 0.01). Additionally, there was no statistically significant correlation between the percentage change in talar tilt and the change in the LDTA.

**Figure 3:**
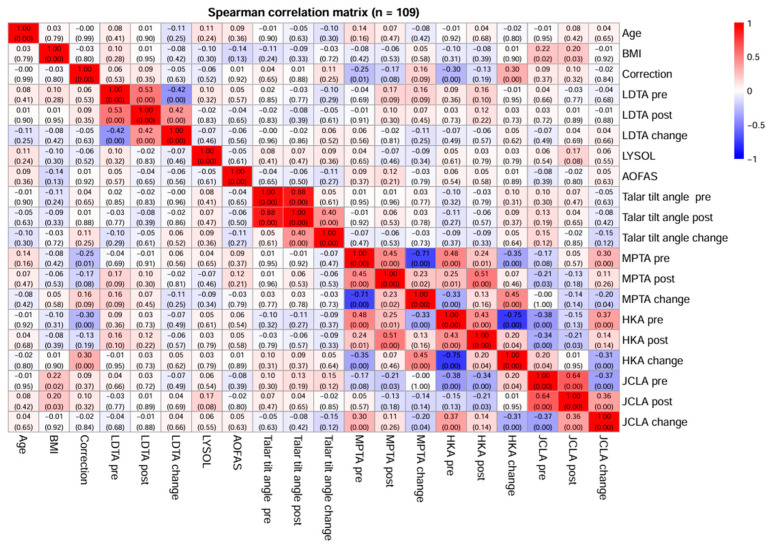
Correlation matrix plot between LYSHOLM and AOFAS scores and radiological measurements (LDTA, HKA, MPTA, JCLA, talar tilt) in the study sample.

No statistically significant relationships were found between the amount of correction and the other radiological parameters (Figure 3).

## 4. Discussion

The most important finding of this study is that it revealed that a MOWHTO resulted in significant improvements not only in knee alignment but also in the alignment of the ankle plane. Statistically significant changes observed in the LDTA and talar tilt angles indicate that the ankle joint line tended toward a configuration that was more parallel to the ground and that the change in the mechanical axis was reflected in distal joint alignment. This can be interpreted as a radiological alignment adaptation. There is increasing evidence that such corrective surgeries not only alter the mechanical axis of the knee but can also reload the ankle joint, thereby carrying the risk of functional improvement or new symptoms [16]. Similar results were reported by Choi et al., who showed that after an HTO, the ankle joint surface tended to shift into valgus, talar tilt decreased, and ankle pain improved [14]. Takeuchi et al. also noted significant reductions in both knee and ankle pain after an HTO and observed that the distal tibial joint surface shifted into valgus [12]. In the present study, a significant reduction in the talar tilt angle was similarly observed, supporting the notion that flattening in the distal joint line was indicated. The changes observed in the ankle joint can be explained by alterations in the tibiotalar contact surface and pressure distribution due to lateralization of the mechanical axis after an HTO. In a cadaveric study by Suero et al., it was demonstrated that HTO corrections of 10–15° reduced the tibiotalar contact surface area and altered intra-articular ankle pressures [17]. Furthermore, this study observed statistically significant improvements in MPTA, HKA, and JLCA parameters. The concurrent flattening seen in the LDTA and talar tilt angles of the ankle joint reinforces the idea that mechanical axis correction influences the distal joint plane. A systematic review by Van Oevelen et al. emphasized that changes in tibio-talar contact and load transfer after an osteotomy—especially in cases of major deformities—can affect ankle biomechanics [1].

The flattening observed in the ankle plane after an HTO may also be partly attributed to the compensatory effects on the subtalar joint. Kazemi et al. reported that the subtalar joint compensates for varus knee deformity by shifting into valgus, and that this tendency diminishes following an HTO, leading to improved heel alignment [10]. Moreover, gait analysis studies have shown that midtarsal and subtalar compensation can reverse with surgical correction, normalizing plantar pressure distribution [18].

Some studies have reported worsening clinical symptoms in the ankle joint. Shah et al. observed new-onset ankle pain in approximately 20% of patients and found that this risk was particularly evident when the tibial slope exceeded 10° [19]. Elsayed et al. reported increased ankle pain after an osteotomy, particularly in cases of overcorrection or in cases where talar tilt and tibial slope were significantly altered [20]. In our study, no corrections greater than 14 mm were performed, and all corrections were targeted to the Fujisawa point, which may have prevented excessive valgus overcorrections. This may explain the high AOFAS scores and the lack of significant ankle symptoms observed.

Other studies have also reported clinical improvements in the ankle joint [9,12]. Although our study did not compare pre- and postoperative clinical scores, the 1-year postoperative clinical scores were within an acceptable range. In all procedures, the correction target was planned according to the Fujisawa point (62% lateral tibial plateau), and no case involved correction beyond 14 mm. This suggests that ankle function can be preserved when corrections are made within reasonable limits after osteotomy.

Comparative studies in the literature report that changes in the ankle and subtalar planes after TKA are more pronounced than those following an HTO [1]. Jeong et al. highlighted that valgus changes in the ankle joint were more frequent after TKA and that some patients became symptomatic [21]. Similar to our findings, other studies have also indicated that when the ankle joint line is brought closer to a more physiological plane, symptomatic improvement can be achieved [2]. Although the angular changes at the ankle were small in absolute magnitude, they were considered to be modest alignment adaptations rather than strong biomechanical effects, interpreted within the context of the existing evidence and effect-size findings. However, the literature indicates that the effects of the mechanical axis on the distal joints after a MOWHTO are mostly minimal and that these changes may reflect lower-extremity adaptation processes. In this context, while the small angular differences in our study are not clinically significant, they are valuable in revealing the direction and nature of the potential effects of mechanical axis correction on the ankle plane. Therefore, our findings are interpreted cautiously in terms of biomechanical significance and are considered limited but consistent indicators of adaptation, consistent with the literature [22].

The findings obtained are consistent with the results of similar studies in the existing literature and demonstrate that a MOWHTO should be evaluated not only at the level of the knee but also at the level of the ankle joint [10,13,14].

This study has several limitations. First, due to its retrospective design, there may be data collection and patient follow-up limitations or missing information. Second, only 1-year follow-up results were evaluated; long-term effects and potential degenerative changes were not addressed. Third, the clinical evaluations were based on subjective scoring systems, which may be influenced by individual factors such as patient expectations or daily living conditions. Additionally, the lack of preoperative clinical scores constitutes a limitation of the study, as this prevents direct comparison of preoperative and postoperative clinical outcomes. Ankle joint assessments were limited to radiographic measurements; advanced imaging methods (e.g., CT, MRI) or dynamic analyses (e.g., gait analysis) were not included in the study. Additionally, the absence of measurements related to forefoot and subtalar joint alignment, as well as the fact that follow-up was limited to a single time point (1 year), restricts the evaluation of the long-term clinical effects of the findings. Lastly, although having all surgeries performed by the same surgical team ensures methodological consistency, it may limit the generalizability of the results.

## 5. Conclusions

In this study, a MOWHTO resulted in improvements in knee-joint mechanical alignment and also statistically significant angular changes in the ankle joint plane, suggestive of a multidimensional effect of the procedure on joint alignment of the lower extremity; however, these findings should be supported by larger sample sizes and long-term studies. These differences in LDTA and talar tilt values are noteworthy because they demonstrate the impact of the mechanical axis on the ankle joint; the clinical impact of these changes needs to be evaluated with further, longitudinal studies including pre- and postoperative biomechanic evaluation and gait analysis.

## Figures and Tables

**Table 1:** Demographic data of patients.

Variables		Participants (*n* = 110)
Gender	Male	28 (25.5%)
	Female	82 (74.54%)
Side	Right	46 (41.81%)
	Left	64 (58.2%)
Kellgren–Lawrence	2	36 (32.7%)
	3	71 (64.5%)
	4	3 (2.7%)
Age		52 (11)
Body Mass Index		29.4 (4)
Correction (mm)		10 (2)

Data are presented as median (IQR). Categorical variables reported as frequency (percent).

**Table 2:** Comparison of measurements taken before and 1 year after surgery.

	Baseline	Postop 1 Year	*p*-Value	Effect Size	ICC (95%CI)
LDTA	87.25 (2.76)	88.14 (2.24)	0.018 ^a^	0.225	0.690 (0.577–0.776)
MPTA	83 (4.53)	87.40 (2.71)	<0.001 ^a^	0.838	0.441 (0.277–0.579)
HKA	171.44 ± 3.35	177.11 ± 2.32	<0.001 ^b^	−1.804	0.404 (0.235–0.549)
JLCA	3.18 (1.94)	1.95 (1.12)	<0.001 ^a^	0.798	0.692 (0.580–0.778)
Talar tilt	0.85 (0.51)	0.70 (0.52)	<0.001 ^a^	0.752	0.863 (0.806–0.904)

Data are presented as mean ± SD and median (IQR). Differences between pre-post measurements were analyzed as follows: ^a^ Wilcoxon test, ^b^ paired-sample t-test. MPTA; medial proximal tibial angle, HKA; hip–knee–ankle angle, JLCA; joint line convergence angle, LDTA; lateral distal tibial angle.

**Table 3:** Difference analysis results in terms of clinical measurements with correction (mm) values.

Variables	<10 mm (*n* = 50)	≥10 mm (*n* = 60)	95% CI (Mean Difference)	*p*-Value	Effect Size
Age	52.50 (13)	52 (10)	1.0 (−2.0–4.0)	0.467 ^a^	0.069
BMI	29.15 (4.5)	29.55 (3.5)	−2.34 × 10^−5^ (−1.00–0.90)	0.993 ^a^	0.000
Gender Male Female	9 (18%) 41 (82%)	19 (31.7%)	-	0.156 ^b^	−0.156
Kellgren grade 2 3 4	18 (36%) 30 (60%) 2 (4%)	18 (30%) 41 (68.3%) 1 (1.7%)	-	0.601 ^c^	0.102
AOFAS	100 (3)	100 (10)	−1.68 × 10^−5^ (−8.18 × 10^−6^–3.83 × 10^−5^)	0.861 ^a^	0.017
LYSHOLM	90 (19)	90 (23)	4.15 × 10^−5^ (−3–4)	0.880 ^a^	0.014
JCLA_percent change	−36.26 (26.19)	−37.07 (26.74)	1.129 (−7.850–8.130)	0.785 ^a^	0.026
LDTA_percent change	0.55 (2.46)	0.65 (3.27)	−0.25 (−1.06–0.579)	0.606 ^a^	0.049
Talar tilt_percent_change	−13.03 (14.06)	−9.11 (16.30)	0.020 (−0.01–0.05)	0.204 ^a^	0.121

Data are presented as median (IQR). ^a^ Mann–Whitney U test, ^b^ Yates’s continuity correction test, ^c^ Fisher–Freeman–Halton test (JLCA; joint line convergence angle, LDTA; lateral distal tibial angle.

## Data Availability

The data presented in this study are available on request from the corresponding author due to privacy and ethical restrictions.

## Abbreviations

AOFAS	American Orthopedic Foot and Ankle Society
HKA	Hip–knee–ankle angle
LDTA	Lateral distal tibial angle
JLCA	Joint line convergence angle
MPTA	Medial proximal tibial angle
MOWHTO	Medial open-wedge high tibial osteotomy
OA	Osteoarthritis
TKA	Total knee arthroplasty
